# BMC Nephrology reviewer acknowledgement, 2013

**DOI:** 10.1186/1471-2369-15-22

**Published:** 2014-02-06

**Authors:** Hayley Henderson

**Affiliations:** 1 BioMed Central, Floor 6, 236 Gray's Inn Road, London WC1X 8HB, UK

## Abstract

**Contributing reviewers:**

The editors of BMC Nephrology would like to thank all our reviewers who have contributed to the journal in Volume 13 (2013).

## 

Ali Inal

Turkey

Hanna Abboud

USA

Mario Abbud-Filho

Brazil

Dina Abdellatif

Egypt

Emaad Abdel-Rahman

USA

Alison Abraham

USA

Matthew Abramowitz

USA

Kenneth Abreo

USA

Emad Abu-Assi

Spain

Philip Acott

Canada

Teresa Adragao

Portugal

John Agar

Australia

Varun Agrawal

USA

Thurid Ahlenstiel

Germany

Farrokhlagha Ahmadi

Iran

Harun Akar

Turkey

Tasuku Akiyama

USA

Ziyad Al-Aly

USA

Mark Dominik Alscher

Germany

Csaba Ambrus

Hungary

Ryoichi Ando

Japan

Tsugunobu Andoh

Japan

Michele Andreucci

Italy

Elio Antonucci

Italy

Shin-Ichi Araki

Japan

Javier Arrieta

Spain

Nishkantha Arulkumaran

UK

Brad Astor

USA

Daniel Athanazio

Brazil

Titus Augustine

UK

Harold Aukema

Canada

Petar Avramovski

Macedonia

Nurettin Aydogdu

Turkey

Parisa Badiee

Iran

Xueyuan Bai

China

Omran Bakoush

Sweden

Jozsef Balla

Hungary

Karen Bandeen-Roche

USA

Debasish Banerjee

UK

Giuseppe Banfi

Italy

Peter Barany

Sweden

Jonathan Barasch

USA

Gina-Marie Barletta

USA

Jonathan Barratt

UK

Pasqual Barretti

Brazil

Cédric Baumann

France

George Bayliss

USA

Ilia Beberashvili

Israel

Kevin Becker

USA

Joachim Beige

Germany

Antonio Bellasi

Italy

Vincenzo Bellizzi

Italy

Paul Bennett

Australia

Enrico Bergamaschi

Italy

Raoul Bergner

Germany

Laureline Berthelot

France

Marnie Bertolet

USA

Dirk Bethe

Germany

Michiel Betjes

Netherlands

Sebastjan Bevc

UK

Axel Bex

Netherlands

Ishir Bhan

USA

Sunil Bhandari

UK

Markus Bickel

Germany

Jonas Bjork

Sweden

Geoffrey Block

USA

Clemens Bockmeyer

Germany

Jean-Jacques Boffa

France

Radovan Bogdanovic

Serbia

Karol Bomsztyk

USA

Ann Bonner

Australia

Peter Boor

Germany

Maurizio Bossola

Italy

Casper P. Bots

Netherlands

Josée Bouchard

Canada

Maxime Bouchard

Canada

Catherine Bouman

Netherlands

Eoin Brennan

Ireland

Sergey Brodsky

USA

Ellen Brooks

USA

Godela Brosnahan

USA

Gemma Browne

Ireland

Nathan Bryan

USA

Hendrik Buikema

Netherlands

Emmanuel Burdmann

Brazil

Stephane Burtey

France

David Calfee

USA

Paolo Calzavacca

Italy

Joiza Lins Camargo

Brazil

Kathleen Campbell

USA

Benjamin Canales

USA

Bernard Canaud

France

Pietro Canetta

USA

Angela Cannas

Italy

Diego Cantarovich

France

Changchun Cao

China

Jacqueline Caramori

Brazil

Miguel Carrasco

Spain

Susana Carrasco

Brazil

Naima Carter-Monroe

USA

Kerri Cavanaugh

USA

Paul Chamney

UK

Alex Chang

USA

Jer-Ming Chang

Taiwan

Arlene Chapman

USA

Jonathan Chemouny

France

Jinn-Yang Chen

Taiwan

Szu-Chia Chen

Taiwan

Teresa Chen

USA

Wei Chen

China

Zhong Chen

China

Gil Chernin

Israel

Ho Jun Chin

Korea, South

Archil Chkhotua

Georgia

Bum Soon Choi

Korea, South

Che-Yi Chou

Taiwan

Richard Chudleigh

UK

Philip Clayton

Australia

Aldo Clerico

Italy

Christa Cobbaert

Netherlands

Steven Coca

USA

Scott Cohen

USA

Giuseppe Conte

Italy

Giuseppe Coppolino

Italy

Tom Cornelis

Netherlands

Giuliana Cortese

Italy

Cécile Couchoud

France

Marie Courbebaisse

France

Adrian Covic

Romania

Emanuele Cozzi

Italy

Deidra Crews

USA

Wenpeng Cui

China

Zhao Cui

China

Adamasco Cupisti

Italy

Marica Cutajar

UK

Neera Dahl

USA

Christoph Daniel

Gibraltar

Michael Darmon

France

Subir Kumar

Das India

Indranil Dasgupta

UK

Simin Dashti

Iraq

Andrew Davenport

UK

Mariza De Andrade

USA

Ian De Boer

USA

Andreana De Mauri

Italy

Johan De Meester

Belgium

Natale De Santo

Italy

Sophie De Seigneux

Switzerland

Sacha De Serres

Canada

Jeroen KJ Deegens

Netherlands

Serpil Muge Deger

Turkey

Arnaud Del Bello

France

Pierre Delanaye

Belgium

Boriana Deliyska

Bulgaria

Christian Delles

UK

Vimal Derebail

USA

Giulia Dessi

Italy

Patrick D'Haese

Belgium

Agostino Di Ciaula

Italy

Javier Diez

Spain

Ljubica Djukanovic

Serbia

Jun-Young Do

Korea, South

Mark Dockrell

UK

Kent Doi

Japan

Jie Dong

China

Xinzhong Dong

USA

Neville Dossabhoy

USA

Caigan Du

Canada

Soner Duman

Turkey

Ali Duzova

Turkey

Hang-Korng Ea

France

Alberto Edefonti

Italy

Somchai Eiam-Ong

Thailand

A. Ahsan Ejaz

USA

Hampus Eklof

Sweden

Theodoros Eleftheriadis

Greece

Timothy Ellam

UK

Kathrin Eller

Austria

Manal Elshamaa

Egypt

Zoltan Endre

Australia

Eric Engels

USA

Ricardo Enríquez

Spain

Erland Erlandsen

Denmark

Pedro Errasti

Spain

F. Fevzi Ersoy

Turkey

Ciro Esposito

Italy

Pasquale Esposito

Italy

Vittoria Esposito

Italy

Michelle Estrella

USA

Dean Eurich

Canada

Joseph Eustace

Ireland

Pieter Evenepoel

Belgium

Eric Fãraille

Switzerland

Fabio Fabbian

Italy

Fadi Fakhouri

France

Mohammad Kazem Fallahzadeh Abarghouei

Iran

Stanley Fan

UK

Robert Fassett

Australia

Micha Feld

Germany

Leonid Feldman

Israel

Ana Elizabeth Figueiredo

Brazil

Kevin Finkel

USA

Martin Flamant

France

Daniele Focosi

Italy

Damian Fogarty

UK

Annalisa Foschi

Italy

Costas Fourtounas

Greece

Chester Fox

USA

Nora Franceschini

USA

Malou Friederich Persson

Sweden

Luc Frimat

France

Robert Frithiof

Sweden

Ping Fu

China

Toshiki Fukui

Japan

Luciana Furci

Italy

Carlo Gaillard

Netherlands

Andrea Galassi

Italy

Daniel Gale

UK

Maurizio Gallieni

Italy

Frances Game

UK

Santiago Garcia

USA

Jocelyn Garland

Canada

Adam Gaweda

USA

Etienne Gayat

France

Simonetta Genovesi

Italy

Reza Ghavamian

USA

Alaleh Gheissari

Iran

Christoforos Giannaki

Cyprus

Maddalena Gigante

Italy

Adriana Girardi

Brazil

Berenice Gitomer

USA

Sileno Giuseppe

Italy

Arda Goceroglu

Netherlands

David Goldsmith

UK

Anderson Gonçalves

Brazil

Emilio Gonzalez-Parra

Spain

Paul Goodyer

Canada

Dimitrios Goumenos

Greece

Jamie Green

USA

Mario Gregori

Italy

Matthew Griffin

Ireland

Konstadina Griva

Singapore

Fabrizio Grosjean

Italy

Andrew Grulich

Australia

Dominique Guerrot

France

Ankur Gupta

India

Eduardo Gutierrez

Spain

Orlando Gutierrez

USA

Benjamin Haaland

Singapore

Michael Haase

Germany

Anja Haase-Fielitz

Germany

Juliette Hadchouel

France

Andrew Hall

Switzerland

Hermann Haller

Germany

Kenneth Hallows

USA

Takayuki Hamano

Japan

Seung Hyeok Han

Korea, South

Garry Handelman

USA

Anke Hannemann

Germany

Borje Haraldsson

Sweden

Kamal Hassan

Israel

Pippa Hawes

UK

Martinez Ramirez Hector Ramon

Mexico

Saskia Heeringa

Switzerland

Gunnar Heine

Germany

Anne-Elisabeth Heng

France

Dagmara Hering

Australia

Leal Herlitz

USA

Guillermo Herrera

USA

Samuel Heyman

Israel

Nicole Hindman

USA

Swapnil Hiremath

Canada

Yuji Hirowatari

Japan

Julia Hofstra

Netherlands

Radovan Hojs

Slovenia

Stefan Holdenrieder

Germany

Patrick Honore

Belgium

Ewout Hoorn

Netherlands

Pascal Houillier

France

Martin Howell

Australia

Tomasz Hryszko

Poland

Chi-Yuan Hsu

USA

Chiu-Ching Huang

Taiwan

Jin-An Huang

Taiwan

Michael Hultström

Sweden

Kuan-Yu Hung

Taiwan

Ender Hur

Turkey

Alastair Hutchison

UK

Shang-Jyh Hwang

Taiwan

Meritxell Ibernon

Spain

Masahiro Ikeda

Japan

Ryota Ikee

Japan

Enyu Imai

Japan

Olafur Skuli Indridason

Iceland

Hiroyuki Inoshita

Japan

Maria Irazabal

USA

Berend Isermann

Germany

Kenta Ito

Japan

Peter Ivanovich

USA

Masayuki Iyoda

Japan

Liotier Jerome

French Polynesia

Navin Jaipaul

USA

Michael Janech

USA

George Jarad

USA

Vanita Jassal

Canada

Guillaume Jean

France

Bente Jespersen

Denmark

Saleem Jessani

Pakistan

Vivekanand Jha

India

Lars Jødal

Denmark

Kirsten Johansen

USA

David Johnson

Australia

Heather Johnson

USA

Vanda Jorgetti

Brazil

Noemie Jourde-Chiche

France

Anna Jovanovich

USA

Theodora Kafkia

Greece

Ayeesha Kamran Kamal

Pakistan

Shinya Kaname

Japan

Johji Kato

Japan

Hiroo Kawarazaki

Japan

Kubra Kaynar

Turkey

Amir Kazory

USA

Mira Keddis

USA

Frieder Keller

Germany

Jessica Kendrick

USA

Andre P Kengne

Cameroon

Mohammad Reza

Khatami Iran

Jan Kielstein

Germany

Jan Kielstein

USA

Yon Su Kim

Korea, South

Susan Kirk

UK

Chagriya Kitiyakara

Thailand

Scott Klarenbach

Canada

Bojan Knap

Slovenia

Felix Knauf

Germany

Bertrand Knebelmann

France

Mladen Knotek

Croatia

Hiroyuki Kobori

USA

Gülay Koçak

Turkey

Paulo Koch Nogueira

Brazil

Ismail Kocyigit

Turkey

Wouter Kok

Netherlands

Akatsuki Kokaze

Japan

Atsushi Komatsuda

Japan

Paul Komenda

Canada

Judith Kooiman

Netherlands

Marion Koopman

Netherlands

Jeffrey Kopp

USA

Pelagia Koufaki

UK

Jay Koyner

USA

Susan Kralisch-Jäcklein

Germany

Andrea Kramer

Netherlands

Matthew Krasowski

USA

Jan Krikken

Netherlands

Zipporah Krishnasami

USA

Thilo Krueger

Germany

Marcin Kruszewski

Poland

Seyhun Kursat

Turkey

Thimo Kurz

UK

Eiji Kusano

Japan

Vivek Kute

India

Vytautas Kuzminskis

Lithuania

Gaetano La Manna

Italy

Helen Lachmann

UK

Mark Lal

Sweden

Susanne Lang

Germany

Vijay Lapsia

USA

Anders Larsson

Sweden

Nicola Latronico

Italy

William Lawson

USA

Colleen Le Prell

USA

David Leaf

USA

Viviane Leal

Brazil

Anne Lee

Denmark

Pauline Lee

USA

Vincent Lee

Australia

Renata Leitao

Brazil

Annette Lennerling

Sweden

Ryan Lennon

USA

Nathan Levin

USA

Joshua Lewis

Australia

Karl Lhotta

Austria

Tingting Li

USA

Gioacchino Li Cavoli

Italy

Kelly Liang

USA

Fernando Liaño

Spain

Helen Liapis

USA

John Lieske

USA

Su-Chi Lim

Singapore

Helbert Lima

Brazil

Bengt Lindholm

Sweden

Gregor Lindner

Switzerland

Mark Little

Ireland

Francesco Locatelli

Italy

Poay Huan Loh

UK

James Lohr

USA

David Long

UK

Jose Antonio Lopes

Portugal

Jose Lopes De Faria

Brazil

Manuel López-Cabrera

Spain

Luca Andrea Lotta

Italy

Jicheng Lv

China

Umberto Maggiore

Italy

Susan VM Maharaj

USA

Mitra Mahdavi-Mazdeh

Iran

Rahul Mainra

Canada

Irena Makulska

Poland

Rahul Malhotra

Singapore

Francesca Mallamaci

Italy

Jolanta Malyszko

Poland

Nseka Mangani Congo

Democratic Republic

Filippo Mangione

Italy

Jacek Manitius

Poland

Carlo Manno

Italy

Piero Marchetti

Italy

Christophe Mariat

France

Saugar Maripuri

USA

Patrick Mark

UK

Pablo Maroto

Spain

Pierre-Yves Martin

Switzerland

Stefania Marzocco

Italy

Anthony Mathelier

Canada

Valeria Matta

Italy

Kim Mcfann

USA

Gareth Mckay

UK

Gearoid Mcmahon

USA

Lawrence Mcmahon

Australia

Mara Medeiros

Mexico

Arianeb Mehrabi

Germany

Esther Meijer

Netherlands

Bjorn Meijers

Belgium

Christa Meisinger

Germany

Michal Melamed

USA

Cathy Mendelsohn

USA

David Mendelssohn

Canada

Claudia Menzaghi

Italy

Mario Meola

Italy

Michael Merchant

USA

Piergiorgio Messa

Italy

Marie Metzger

France

Renzo Mignani

Italy

Francis Miller

USA

Akira Mima

Japan

Roberto Minutolo

Italy

Donald Molony

USA

Karen Molyneux

UK

Santo Morabito

Italy

Juan Moreno

Spain

Matthew Morgan

UK

Yoshiyuki Morishita

Japan

Takahito Moriyama

Japan

Mojgan Mortazavi

Iran

Rachael Morton

Australia

Amy Mottl

USA

David Mount

USA

Ivan Moura

France

Rosa Moyses

Brazil

Phillip Mucksavage

USA

Bruce Mueller

USA

Giuseppe Mule'

Italy

Corrado Murtas

Italy

Girish Nadkarni

USA

Yasuyuki Nagasawa

Japan

Kimimasa Nakabayashi

Japan

Hitoshi Nakashima

Japan

Masaru Nakayama

Japan

Serafeim Nanas

Greece

Cuneyt Narin

Turkey

Andrew Narva

USA

Sankar Navaneethan

USA

Armando Negri

Argentina

Guy Neild

UK

Susanne Nicholas

USA

Gian Luigi Nicolosi

Italy

Zofia Niemir

Poland

Amiram Nir

Israel

Ravi Nistala

USA

Dorothea Nitsch

UK

Marlies Noordzij

Netherlands

Keith Norris

USA

Shaikh Nurmohamed

Netherlands

Yoshitsugu Obi

Japan

Aghogho Odudu

UK

William Oetting

USA

Norma Ofsthun

USA

Makoto Ogura

Japan

Kook-Hwan Oh

Korea, South

Naro Ohashi

Japan

Mitsuru Ohishi

Japan

Takayasu Ohtake

Japan

Rieko Okada

Japan

Ikechi Okpechi

South Africa

Rik Olde Engberink

Netherlands

Albert Ong

UK

Macaulay Onuigbo

USA

Shahrzad Ossareh

Iran

Marlies Ostermann

UK

Alfonso Otero Gonzalez

Spain

Christian Ott

Germany

Islem Ouanes

Tunisia

Heleen M Oudemans-Van Straaten

Netherlands

Sandosh Padmanabhan

UK

Amir Pakpour

Iran

Alberto Palazzuoli

Italy

Nicolas Pallet

France

Antonello Pani

Italy

Ernesto Paoletti

Italy

Eugenia Papakrivopoulou

UK

Rulan Parekh

Canada

Nisha Parikh

USA

Mark Parker

USA

Rashmi Patel

India

Samir Patel

USA

Vishal Patel

USA

Sandipan Pati

USA

Lars Penne

Netherlands

Ruth Pepper

UK

Mark Perazella

USA

Jazmin Perez

Mexico

Miguel Perez Fontan

Spain

Giovanni Pertosa

Italy

Francesco Pesce

UK

Marcus Pezzolesi

USA

Phuong-Thu Pham

USA

Barbara Philips

UK

Maria Picken

USA

Laura Plantinga

USA

Victor Pollak

USA

Rafael Ponikvar

Slovenia

Roberto Pontremoli

Italy

Frank Post

UK

Hans Pottel

Belgium

Kearkiat Praditpornsilpa

Thailand

Jai Prakash

India

Giuseppe Procopio

Italy

Thyago Proenca De Moraes

Brazil

Giuseppe Pugliese

Italy

Patrick Pun

USA

Wajeh Qunibi

USA

Rapur Ram

India

Mahboob Rahman

USA

Shamima Rahman

UK

Luca Rampoldi

Italy

Gopi Rangan

Australia

Richard Ransom

USA

Effat Razeghi

Iran

Giuseppe Regolisti

Italy

Jochen Reiser

USA

Jana Reiterova

Czech Republic

Giuseppe Remuzzi

Italy

Marc Rendell

USA

Ana Ricardo

USA

Dena Rifkin

USA

Stephen Riley

UK

Cassianne Robinson-Cohen

USA

Michael Robson

UK

Roger Rodby

USA

Jose C. Rodriguez-Perez

Spain

Nicolas Rognant

France

Lodi Roksnoer

Netherlands

Marcel Roos

Germany

Steven Rosansky

USA

Alexander Rosenkranz

Austria

Peter Rossing

Denmark

Shiva Roy

Australia

Isabelle Rubera

France

Dvora Rubinger

Israel

Tim Rudd

UK

Charumathi Sabanayagam

Singapore

Massimo Sabbatini

Italy

Alaa Sabry

Egypt

Mohammad Mahdi Sagheb

Iran

Johan Sällström

Sweden

Ramin Sam

USA

Leyla Sancho

Brazil

Richard Sandford

UK

Simone Sanna-Cherchi

USA

Pantelis Sarafidis

Greece

Giovanna Sau

Italy

Patrick Saudan

Switzerland

Joost Schanstra

France

Francesco Paolo Schena

Italy

Helmut Schiffl

Germany

Raphael Schiffmann

USA

Holger Schmid

Germany

Richard Schmidt

USA

Antoine Schneider

Switzerland

John Schollum

New Zealand

Morten Schou

Denmark

Julia Scialla

USA

Francesco Scolari

Italy

Yoav Segal

USA

Antonio Seguro

Brazil

Nicholas Selby

UK

Cristina Sena

Portugal

Tariq Shafi

USA

Sanjeev Shah

Uruguay

Vahakn Shahinian

USA

Deep Sharma

USA

James Sharman

Australia

Catriona Shaw

UK

Neil Sheerin

UK

Michiko Shimada

Japan

Tatsuo Shimosawa

Japan

Ho Sik Shin

Korea, South

Sug Kyun Shin

Korea, South

Theresa Shireman

USA

Sandra Silveiro

Brazil

Justin Silver

Israel

Donald Silverberg

Israel

Ismail Simsek

Turkey

Manish Sinha

UK

Suthesh Sivapalaratnam

Netherlands

Hicham Skali

USA

Amy Skversky

USA

Alice C Smith

UK

Joanna Smith

UK

Roy L. Soiza

UK

Amy Sood

Canada

Jacob Sosna

Israel

Kevin Sowinski

USA

Stephen Sozio

USA

Nattachai Srisawat

USA

Austin Stack

Ireland

Anthony Staines

Ireland

Andréa Stinghen

Brazil

Tomasz Stompor

Poland

Christos Stournaras

Greece

Stephanie Stringer

UK

Giovanni Strippoli

Italy

Dirk Struijk

Netherlands

Kostas Stylianou

Greece

Hitoshi Sugiyama

Japan

Kumar Sujeet

USA

Jennfier Sullivan

USA

Ernest Sumaili Congo

Democratic Republic

Angela Summers

UK

Roger Sur

USA

Barbara Suwelack

Germany

Cheuk Chun Szeto

Hong Kong

Silke Szymczak

USA

Eiji Takeda

Japan

Tsukasa Takemura

Japan

Frederick Tam

UK

Ying-Cai Tan

USA

Navdeep Tangri

Canada

Hamid Tayebi Khosroshahi

Iran

Ugo Teatini

Italy

Boon Wee Teo

Singapore

Hiroyuki Terawaki

Japan

Vladimir Tesar

Czech Republic

Lehana Thabane

Canada

Charuhas Thakar

USA

Sumeska Thavarajah

USA

Fernando Thomé

Brazil

Robin Thorpe

UK

Adrienne Tin

USA

Francesca Tinti

Italy

Stephanie Titze

Germany

Marcin Tkaczyk

Poland

Yoshiyuki Tohno

Thailand

Marcello Tonelli

Canada

Allison Tong

Australia

Nicholas Topley

UK

Roser Torra

Spain

Massimo Torreggiani

Italy

Vasilis Tsimihodimos

Greece

Nobuo Tsuboi

Japan

Hiroyasu Tsukaguchi

Japan

Delphine Tuot

USA

Claire Turner

UK

Seiji Ueda

Japan

Tim Ulinski

France

Pablo Antonio Urena Torres

France

Giovanna Valenti

Italy

Tom Van Agtmael

UK

Marjolijn Van Buren

Netherlands

Peter Van Buren

USA

Bert-Jan Van Den Born

Netherlands

Jan Van Den Brand

Netherlands

Antonie Van Den Meiracker

Netherlands

Frank Van Der Sande

Netherlands

Barbara Van Munster

Netherlands

Gijs Van Pottelbergh

Belgium

Priya Vart

Netherlands

Nosratola D. Vaziri

USA

Joachim Velden

Germany

Joseph Verbalis

USA

Francisco Veronese

Brazil

Agnes Veyradier

France

Jose Vieira, Jr.

Brazil

Volker Vielhauer

Germany

Cecil Vigneau

France

Giuseppe Villa

Italy

Guillermo Villa

Spain

Liffert Vogt

Netherlands

Edward Vonesh

USA

Hani Wadei

USA

Ayman Wahbeh

Jordan

Rob Walker

New Zealand

Stephen Walsh

UK

Jiguang Wang

China

Wen Wang

UK

Yin Wang

USA

Louise Watson

UK

Andrew Webb

UK

Daniel Weiner

USA

Eric Weinhandl

USA

Thomas Weinreich

Germany

Matthew Weir

USA

Steven Weisbord

USA

David Weisman

USA

Chi Pang Wen

Taiwan

Christof Westenfelder

USA

Gillian Whalley

New Zealand

Sarah White

USA

William Whittier

USA

Larysa Wickman

USA

Kathryn Wiggins

Australia

Eranga Wijewickrama

Sri Lanka

Martin Wilkie

UK

Craig Wong

USA

Germaine Wong

Australia

James Wright

Canada

Ming-Ju Wu

Taiwan

Peng-Cheng Xu

China

Yu Xu

China

Kunihiro Yamagata

Japan

Takashi Yasuda

Japan

Jerry Yee

USA

Chih-Ching Yeh

Taiwan

Mahmut Ilker Yilmaz

Turkey

Mauricio Younes-Ibrahim

Brazil

Doaa Youssef

Egypt

Luis Yu

Brazil

Peter Yuen

USA

Stuart Yuspa

USA

Nadia Zalunardo

Canada

Tomasz Zapolski

Poland

Donatella Zappulla

Italy

Luxia Zhang

China

Minghui Zhao

China

Fude Zhou

China

Fansan Zhu

USA

Emanuel Zitt

Austria

Carmine Zoccali

Italy

Li Zuo

China

Fable Zustovich

Italy

## Pre-publication history

The pre-publication history for this paper can be accessed here:

http://www.biomedcentral.com/1471-2369/15/22/prepub

